# Disseminated Histoplasmosis Involving Soft Palate, Duodenum, Sigmoid Colon and Bone Marrow in a Patient With Isolated CD4+ T-Lymphocytopenia

**DOI:** 10.7759/cureus.19748

**Published:** 2021-11-19

**Authors:** Naga Swetha Samji, Rajanshu Verma, Sanobar Y Mohammed, Farhan Khan, Mohammad K Ismail

**Affiliations:** 1 Internal Medicine, Tennova Cleveland Hospital, Cleveland, USA; 2 Gastroenterology, University of Tennessee Health Sciences Center, Memphis, USA; 3 Pathology and Laboratory Medicine, University of Tennessee Health Sciences Center, Memphis, USA

**Keywords:** isolated cd4 lymphocytopenia, rare disease, immunology, lymphocytopenia, idiopathic cd4 lymphocytopenia, disseminated histoplasmosis

## Abstract

Idiopathic CD4 T-lymphocytopenia (ICL) is a rare entity that is associated with decreased immunity which predisposes affected individuals to opportunistic infections and malignancies. Autoimmune conditions are common in patients with ICL and they are considered part of its clinical spectrum as well. Treatment of ICL includes treatment of opportunistic infections and prophylaxis against them. Some cases are self-limited while others require long-term monitoring. We present a case of a 60-year-old man who was diagnosed with disseminated histoplasmosis involving soft palate, duodenum, colon and bone marrow in the setting of idiopathic CD4 T-lymphocytopenia.

## Introduction

Idiopathic CD4 T-lymphocytopenia (ICL) is a rare syndrome associated with various opportunistic infections, autoimmune diseases and neoplastic conditions which was first described by CDC in 1992 [1]. Diagnosis of ICL is made when low CD4 counts are detected on two separate occasions in the absence of human immunodeficiency virus (HIV) or other CD4-suppressing conditions. Since then, approximately 500 cases have been reported so far. Its treatment paradigms have been borrowed from HIV as no randomized controlled trials exist on ICL and most data has been obtained from case reports and retrospective studies.

This article was previously presented as a meeting poster at the 2021 College of American Pathologists (CAP) Annual Scientific Meeting CAP21 in Chicago on September 25, 2021.

## Case presentation

A 60-year-old heterosexual man presented with 75 lb weight loss, dysphagia, jaw pain/swelling, hypotension and acute kidney injury. Medical history was significant for emphysema, hypertension, stroke, alcohol withdrawal seizure, alcohol abuse and cigarette smoking. He worked as a landscaper.

**Figure 1:**
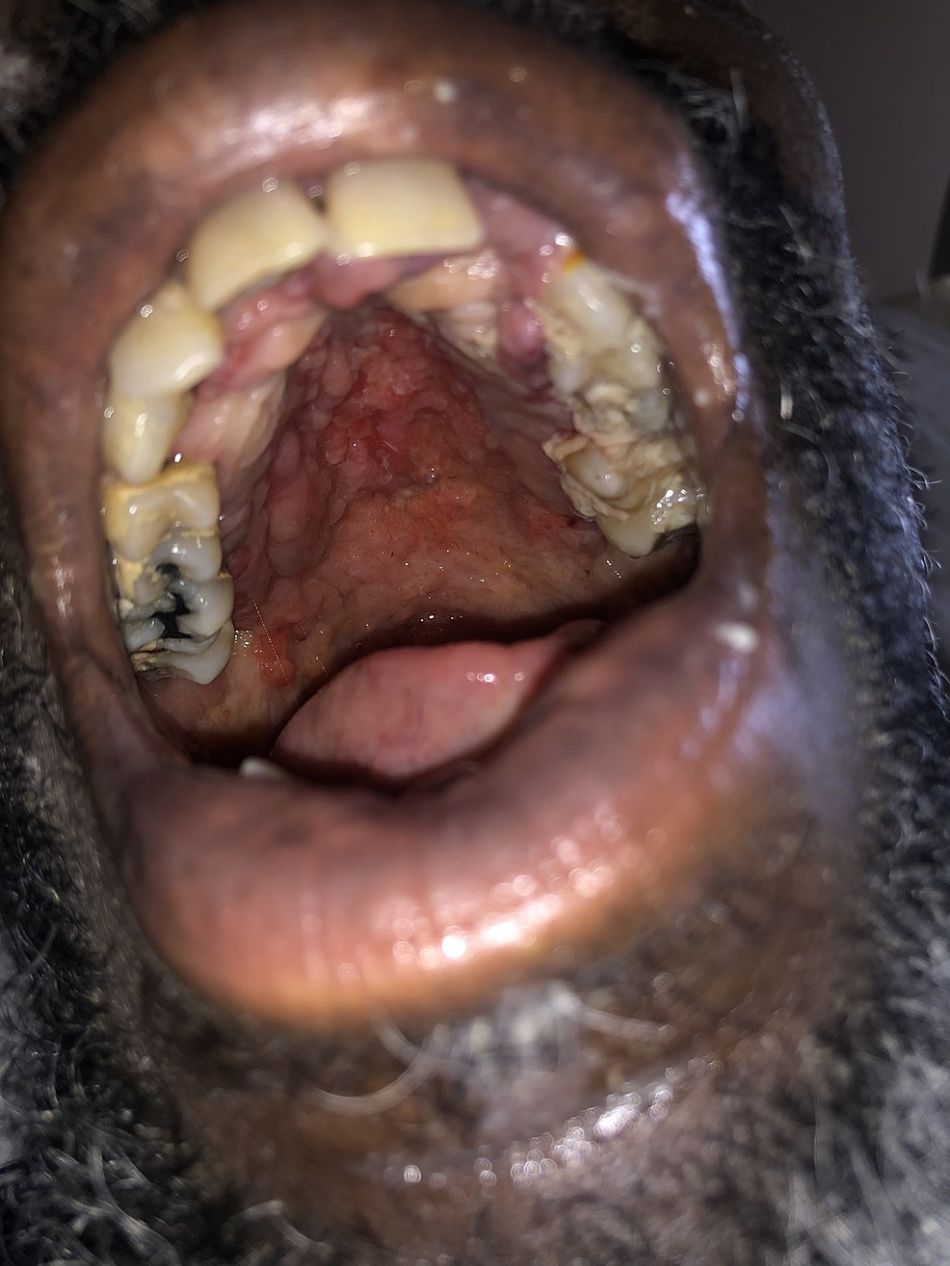
Oral cavity (patient). Hard and soft palate of the patient showing exophytic growth which was found to be a manifestation of disseminated histoplasmosis on biopsy.

On presentation vitals were: 37.5°C, 68/48 mmHg, 95 beats/min, respiratory rate 22/min, oxygen saturation 99% on room air. Physical exam revealed halitosis, poor dentition, diffuse lower gum swelling and erythema. Soft palate showed exophytic growth (Figure 1).

**Figure 2:**
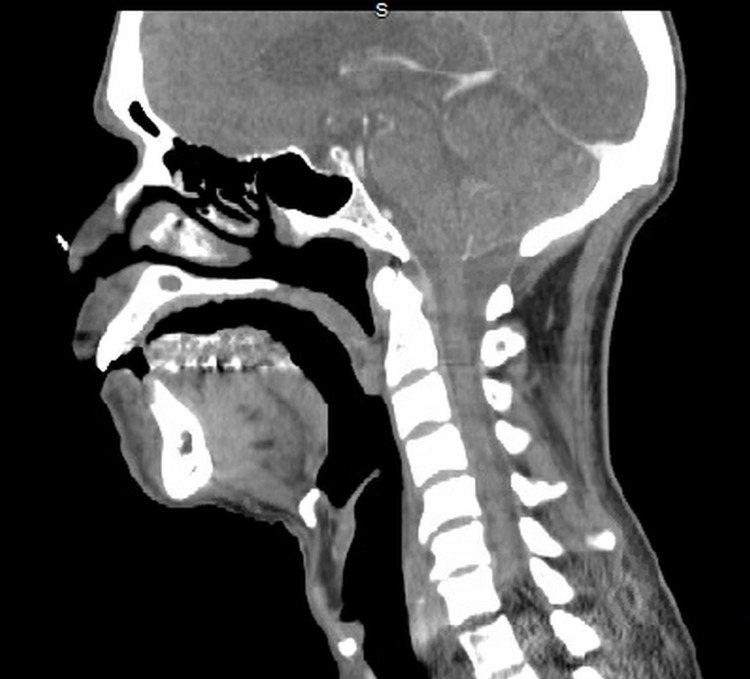
CT scan soft tissue neck (sagittal view) showing soft palate thickening.

Abdominal exam was unremarkable. Serum chemistries showed sodium 139 mEq/L, chloride 102 mEq/L, bicarbonate 30 mEq/L, blood urea nitrogen 43 mg/dl, glucose 94 mg/dl, calcium 8.7 mg/dl, creatinine 2.3 (baseline 1.1 mg/dl), albumin 1.5 g/dl, alkaline phosphatase 574 U/L, lactic acid 2.9 mmol/L, Hb 10.8 g/dl with normal MCV, RDW, decreased RBC 3.87 x 106/ul (normal > 4.2), normal WBC 5000/ul, platelets 167,000/ul, normal bilirubin and ALT. CT neck (Figure 2) showed soft palate asymmetry with thickening.

**Figure 3:**
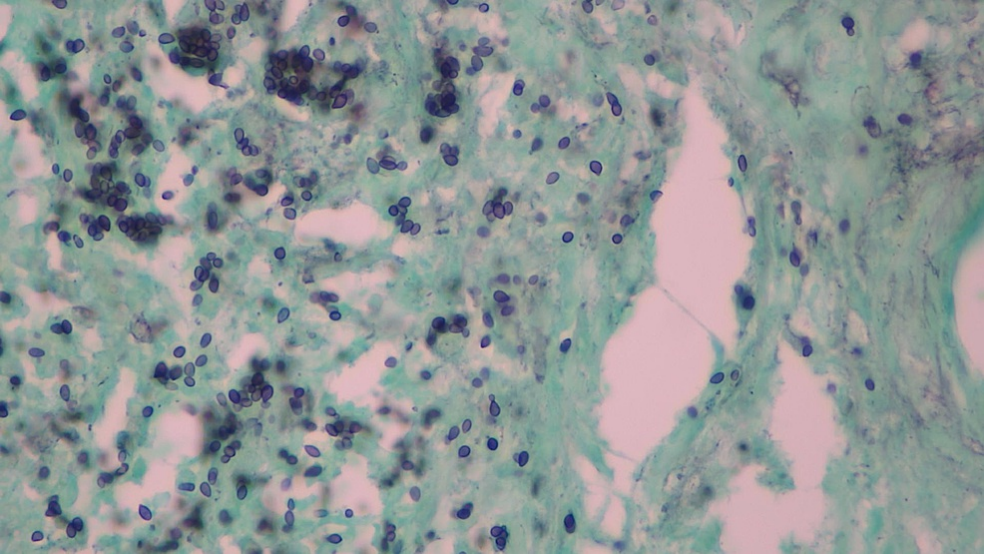
Gomori-Methenamine Silver (GMS) stain (palate biopsy). GMS stain (40x magnification) soft palate with intracellular and extracellular fungal organisms compatible with histoplasma capsulatum.

Piperacillin-tazobactam was started in addition to chlorhexidine empirically to provide coverage for any deep-seated bacterial infection of the oral mucosa while providing anaerobic coverage. ENT did soft palate biopsy which on GMS stain showed numerous round to oval yeast forms present intracellularly within the histiocytes and in the extracellular subepithelial tissue consistent with histoplasmosis (Figure 3).

HIV-1/HIV-2 antigen/antibody screen was negative. Piperacillin-tazobactam was switched to amphotericin B. CD4 count was 36 cells/ul, CD8 count was normal, CD4:CD8 0.1 (normal: 1-3.6). Given a low CD4 count, he was started on azithromycin and sulfamethoxazole-trimethoprim for prophylaxis. Further testing revealed HIV RNA viral load (negative), HTLV-1/HTLV-2 DNA PCR (negative), hepatitis panel testing for hepatitis A, B and C (negative), vitamin B12 (normal), folate 3.7 ng/ml (low), reticulocyte count 2.2%, lactate dehydrogenase (normal), haptoglobin (normal), iron studies showed elevated ferritin, low iron, elevated transferrin saturation (anemia of chronic inflammation), copper (normal), zinc levels 37 mcg/dl (low), SPEP/UPEP (no monoclonal protein; diffuse hypergammaglobulinemia seen), IgA 467 mg/dl (high), IgG 2160 mg/dl (high), IgM 252 mg/dl (high), free light chain ratio (normal). Anti-nuclear antibody (ANA) 1:160, anti-CCP (cyclic citrullinated peptide) IgG 3.3 U/dl (high). Rapid plasma reagin (RPR) (negative), cytomegalovirus PCR (negative) and Epstein-Barr virus PCR were negative. Ultrasound abdomen showed no organomegaly. Bone marrow biopsy showed granulomas consistent with a diagnosis of histoplasmosis.

**Figure 4:**
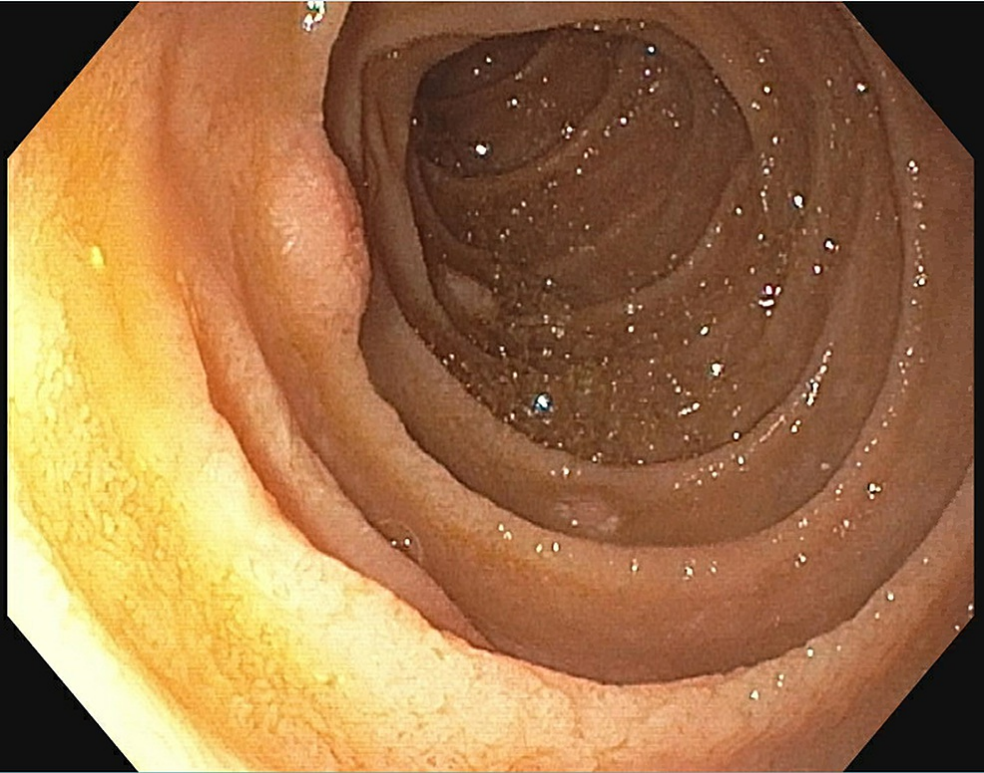
Esophagogastroduodenoscopy (EGD) view - duodenum. Endoscopic view of the second portion of duodenum showing 3-5 mm nodules which on biopsy showed histoplasmosis.

The patient subsequently developed upper gastrointestinal bleeding, so an EGD was performed which showed multiple 3-5 mm nodules in the second part of the duodenum (Figure 4) which were biopsied.

**Figure 5:**
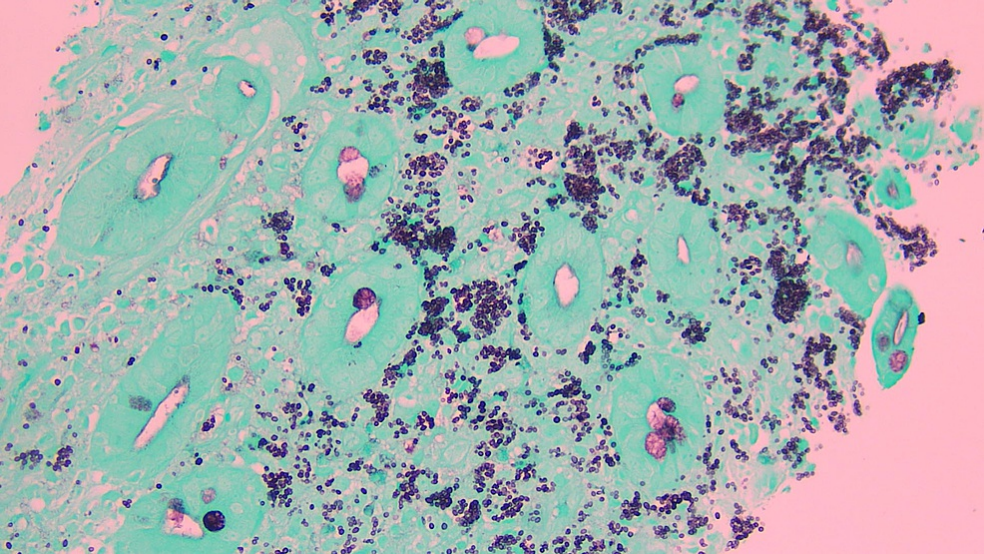
Gomori-Methenamine Silver stain (duodenal biopsies). Gomori Methenamine Silver (GMS) stain (4x magnification) from duodenal biopsies showing duodenitis and highlighting fungal organisms compatible with histoplasmosis.

Pathology showed duodenitis with fungal organisms on Gomorri-Methenamine Silver (GMS) stain compatible with histoplasmosis (Figure 5).

**Figure 6:**
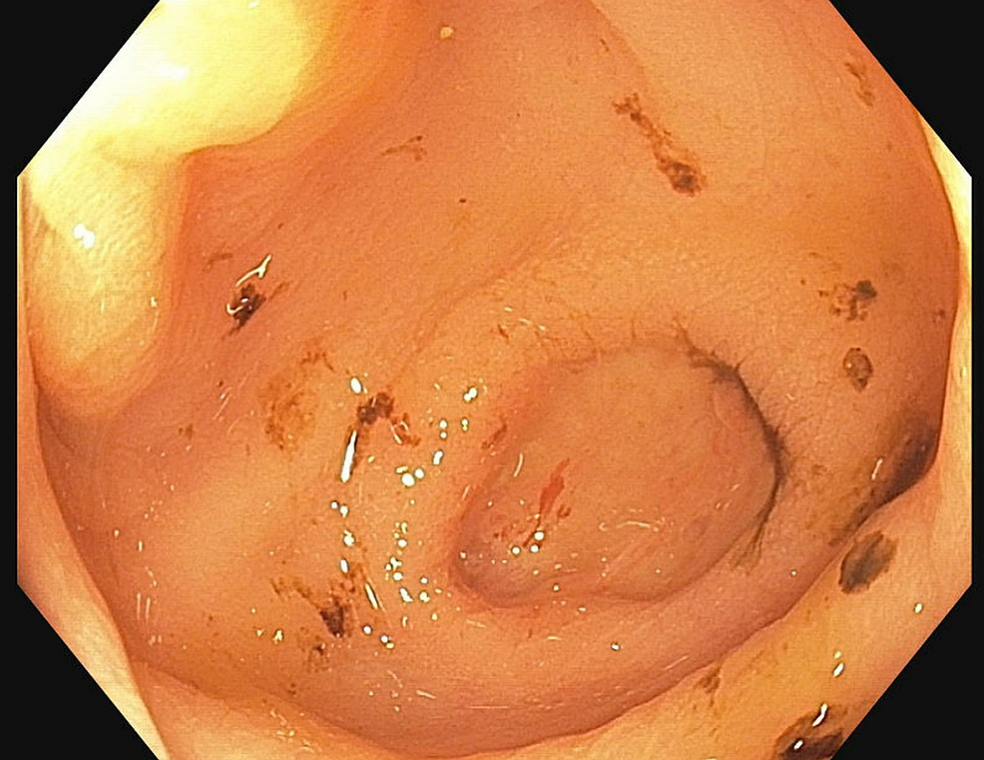
Colonoscopy: endoscopic view - sigmoid colon. Endoscopic view of sigmoid colon showing a 1 cm size clean-based ulcer which was biopsied and showed presence of histoplasma.

**Figure 7:**
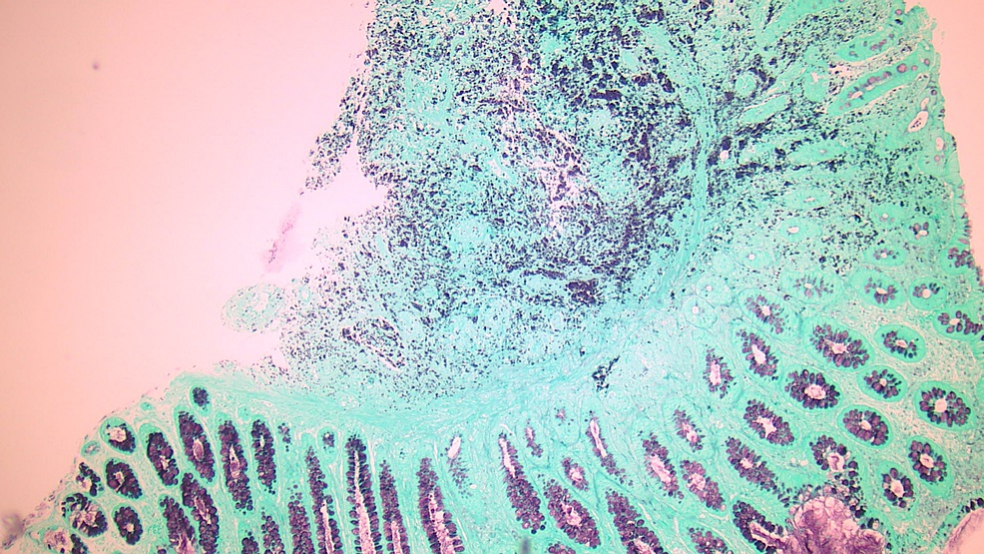
Gomori-Methenamine Silver (GMS) stain - colonoscopy biopsy. Gomori-Methenamine Silver (GMS) stain (4x magnification) showing colonic mucosa with lamina propria highlighting fungal organisms compatible with histoplasma capsulatum infection.

CMV immunostain and AFB stain were negative. Colonoscopy showed diverticulosis, a 1 cm clean-based ulcer in the sigmoid colon (Figure 6) which on biopsy showed the presence of histoplasma (Figure 7).

The patient was then switched to oral itraconazole and discharged home on the same along with azithromycin, sulfamethoxazole-trimethoprim and folic acid.

Four months later, the patient had gained 10 lbs of weight, his dysphagia had resolved, and he was continuing his itraconazole therapy. His CD4 count now was 55/ul. Repeat low CD4+ T-cell count satisfied the criteria for the diagnosis of ICL.

## Discussion

ICL is defined as low CD4+ T-cell count (< 300/mm^3^) or < 20% of total T-cells on two occasions at least six weeks apart in absence of any immunosuppressive state (HIV, other immunodeficiency or medications which decrease CD4 cells) [1]. Up to one-half of ICL patients develop autoantibodies some of which are against CD4 cells [2,3]. Some authors have suggested autoimmune disease to be part of the ICL spectrum [3]. Typically, these patients have normal or low levels of immunoglobulins [4]; however, in our case, immunoglobulins were found to be elevated (possibly secondary to autoimmunity). Spectrum of ICL may vary from an asymptomatic state to life-threatening opportunistic infections. Patients are exposed to myriad opportunistic infections, e.g., cryptococcosis, herpes zoster, recalcitrant warts, candidiasis, progressive multifocal leukoencephalopathy, mycobacterium-avium complex, CMV, viral meningitis including disseminated histoplasmosis [3,5] and up to 20% may develop neoplasms (lymphomas, sarcomas, carcinomas and others) [5,6].

The cause of decreased CD4 cell count in ICL is not known. Various mechanisms such as increased T-cell apoptosis, CD4 sequestration, cytokine dysregulation, gene defects and other etiologies have been implicated [5, 6]. Treatment of ICL revolves around its identification, treatment of and prophylaxis against opportunistic infections. Some authors have suggested treatment with subcutaneous polyethylene glycol-modified-IL-2 injection as well as recombinant human IL-7 to increase the number of circulating CD4 T-cells. Stem cell transplant has also been reported for treating ICL patients with life-threatening infections [7].

Histoplasma is a ubiquitous dimorphic fungus that is present in the soil. Patients are at a higher risk of developing histoplasmosis if the CD4 count is less than 150/ul [8]. Progressive disseminated histoplasmosis (PDH) from gastrointestinal standpoint may be characterized by weight loss, oral ulcers, diarrhea, jaundice, pancytopenia and hepatosplenomegaly [9]. In immunocompromised individuals, PDH may result in abdominal pain, GI bleeding, colonic masses, perianal ulcers, small and large bowel ulcers and perforations [9]. Terminal ileum and cecum are frequently involved [9]. Histoplasma has been detected on autopsy in the GI tract in up to 90% of patients with PDH [10]. In esophagus, erosions, ulcers, traction diverticula, abscesses, tracheoesophageal fistulae may form due to lymph node involvement by histoplasma [10]. Gastric involvement may show ulcers, masses and intestinal involvement is characterized by ulcers, obstruction, strictures and appendicitis [10]. Mesenteric lymphadenopathy has been reported as well [10].

Previously reported cases of histoplasmosis in the setting of ICL mentioned the involvement of skin [11,12], soft palate, lung [12,13], liver, spleen, bone marrow [12,14], and olecranon bursa [11]. All previously reported cases had normal or decreased levels of immunoglobulins [14]. Histoplasmosis can be diagnosed by culture, fungal stains (tissue histochemistry using GMS/Grocott silver stain), serologic tests for antibodies (complement fixation/antibody immunodiffusion) and serum/urine antigen detection. Treatment of disseminated disease is with two weeks of intravenous amphotericin B followed by one year of twice-daily itraconazole.

To the best of our knowledge, this is the first reported case of disseminated histoplasmosis involving the gastrointestinal tract, bone marrow with elevated immunoglobulin levels in a patient with ICL. Gastroenterologists should be cognizant of the myriad GI manifestations of disseminated histoplasmosis.

## Conclusions

This case emphasizes the importance of recognizing ICL as a separate entity and that thorough investigations (as outlined above) should be undertaken to find out the cause of CD4 suppression. In absence of any other cause and repeat testing on another occasion (at least six weeks apart) would allow the reader to make a diagnosis of ICL. ICL just like HIV, can frequently result in opportunistic infections and so the reader should be vigilant regarding identification, screening and prevention of such infections using antimicrobial prophylaxis. Due to its rarity, no randomized control trials are available and management of ICL involves its identification, treatment of and prophylaxis against opportunistic infections.
